# Novel Small-Molecule Troponin Activator Increases Cardiac Contractile Function Without Negative Impact on Energetics

**DOI:** 10.1161/CIRCHEARTFAILURE.121.009195

**Published:** 2021-11-08

**Authors:** Huamei He, Tomas Baka, James Balschi, Alykhan S. Motani, Kathy K. Nguyen, Qingxiang Liu, Rebecca Slater, Brooke Rock, Chen Wang, Christopher Hale, Georgios Karamanlidis, James J. Hartman, Fady I. Malik, Jeff D. Reagan, Ivan Luptak

**Affiliations:** Physiological NMR Core Laboratory, Brigham and Women’s Hospital, Harvard Medical School, Boston, MA (H.H., J.B.).; Institute of Pathophysiology, Faculty of Medicine, Comenius University, Bratislava, Slovakia (T.B.).; Amgen Research, Department of Cardiometabolic Disorders, Amgen Inc, Thousand Oaks, CA (A.S.M., K.K.N., Q.L., R.S., B.R., C.W., C.H., G.K., J.D.R.).; Cytokinetics Inc, South San Francisco, CA (J.J.H., F.I.M.).; Myocardial Biology Unit, Boston University School of Medicine, MA (T.B., I.L.).

**Keywords:** energy metabolism, hemodynamics, phosphocreatine, sarcomeres, troponin

## Abstract

Current heart failure therapies unload the failing heart without targeting the underlying problem of reduced cardiac contractility. Traditional inotropes (ie, calcitropes) stimulate contractility via energetically costly augmentation of calcium cycling and worsen patient survival. A new class of agents—myotropes—activates the sarcomere directly, independent of calcium. We hypothesize that a novel myotrope TA1 increases contractility without the deleterious myocardial energetic impact of a calcitrope dobutamine.

**Methods:**

We determined the effect of TA1 in bovine cardiac myofibrils and human cardiac microtissues, ex vivo in mouse cardiac fibers and in vivo in anesthetized normal rats. Effects of increasing concentrations of TA1 or dobutamine on contractile function, phosphocreatine and ATP concentrations, and ATP production were assessed by ^31^P nuclear magnetic resonance spectroscopy on isolated perfused rat hearts.

**Results:**

TA1 increased the rate of myosin ATPase activity in isolated bovine myofibrils and calcium sensitivity in intact mouse papillary fibers. Contractility increased dose dependently in human cardiac microtissues and in vivo in rats as assessed by echocardiography. In isolated rat hearts, TA1 and dobutamine similarly increased the rate-pressure product. Dobutamine increased both developed pressure and heart rate accompanied by decreased phosphocreatine-to-ATP ratio and decreased free energy of ATP hydrolysis (ΔG_~ATP_) and elevated left ventricular end diastolic pressure. In contrast, the TA1 increased developed pressure without any effect on heart rate, left ventricular end diastolic pressure, phosphocreatine/ATP ratio, or ΔG_~ATP_.

**Conclusions:**

Novel myotrope TA1 increased myocardial contractility by sensitizing the sarcomere to calcium without impairing diastolic function or depleting the cardiac energy reserve. Since energetic depletion negatively correlates with long-term survival, myotropes may represent a superior alternative to traditional inotropes in heart failure management.

## Abstract 2

Supplemental Digital Content is available in the text.

**What Is New?**

- The small-molecule troponin activator (TA1) utilizes a novel mechanism of sarcomeric activation to sensitize the sarcomere to calcium.
- TA1 dose dependently increases contractility ex vivo and in vivo in anesthetized normal rats.
- In isolated hearts, TA1 and the traditional inotrope dobutamine similarly increased rate-pressure product. Dobutamine increased both developed pressure and heart rate accompanied by decreased phosphocreatine-to-ATP ratio and decreased free energy of ATP hydrolysis (ΔG_~ATP_). In contrast, TA1 increased contractile function without negative effects on myocardial energetics.

**What are the Clinical Implications?**

- Current heart failure guideline-directed medical therapies improve myocardial performance by inhibiting components of neurohumoral activation without addressing the underlying problem of decreased cardiac contractility.
- Low blood pressure frequently limits uptitration of guideline-directed medical therapies.
- Traditional inotropes improve cardiac contractility and cardiac output but worsen long-term survival. This deleterious mortality effect had been attributed to excessively increased oxygen consumption and worsening energetics.
- Our data suggest that myotropes may be able to increase cardiac output without negative effects on myocardial energetics.

Currently available treatments for heart failure (HF) with reduced ejection fraction unload the failing heart by blocking components of neurohumoral activation triggered by hypoperfusion of peripheral tissues. The failing heart is known to be energetically starved, and mechanical unloading improves the mismatch between ATP supply and demand.^1^ β-Blockers, ACE (angiotensin-converting enzyme) inhibitors, aldosterone blockers, angiotensin receptor neprilysin inhibitors, and most recently SGLT2 (sodium-glucose linked cotransporter-2) inhibitors have all greatly improved patients’ outcomes in last decades.^2,3^ However, despite these advances, the prognosis for patients with HF remains poor, and ≈75% of patients diagnosed with HF die within 5 years of initial hospitalization, presenting an unmet need.^4^ Before the introduction of β-blockers to chronic HF treatment in 1990’s,^5^ the intuitive opposite approach, to increase the contractile performance, had been used with disappointing results. Despite addressing the main driver of HF, inotropic drugs that acutely improve contractility and cardiac output worsened long-term survival in clinical trials.^2,6,7^ Excessively increased cardiac energetic demand due to increased cAMP signaling and calcium cycling is likely responsible for such adverse outcomes^8^ and is in line with an almost century-old hypothesis that the failing heart is energy starved.^9^ Recently, small-molecule cardiac sarcomere activators that directly increase contractility, that is, myotropes, have been introduced to the research and clinical communities.^10^ Myotropes directly activate cardiac myosin or sensitize the regulatory proteins to calcium without activating cAMP signaling or increasing calcium cycling.^11,12^ However, it is unknown whether myotropes are devoid of the negative effects on the myocardial energetics observed with the traditional inotropes.

Using ^31^P nuclear magnetic resonance (NMR) spectroscopy, we and others have previously shown in different models of heart disease that a mismatch between ATP demand and supply results in accumulation of ADP, decreased phosphocreatine (PCr)/ATP ratio, and decreased ΔG_~ATP_, which is the energy available for ATPases to perform work, that is, cardiac energy reserve.^13–17^ Interventions that rebalance ATP supply/demand mismatch acutely^18^ and chronically^17,19^ improve ΔG_~ATP_, left ventricular (LV) diastolic function, and contractile reserve. Here, we investigated the energetic and functional impacts of the novel small-molecule troponin activator, TA1, and the traditional inotrope, dobutamine (Dob), in normal rat hearts.

We hypothesized that direct activation of the cardiac sarcomere with TA1, in contrast to Dob, would improve cardiac performance without depleting the cardiac energy reserve or worsening of the LV end diastolic pressure (LVEDP). To that end, we demonstrated that TA1 directly increases contractility by enhancing calcium sensitivity and equivalent doses of TA1 augment developed pressure (DevP) more than the traditional inotrope Dob. In contrast to Dob, TA1 does not worsen cardiac energetics (PCr/ATP and ΔG_~ATP_) nor it raises diastolic pressure or heart rate (HR). Moreover, magnetization transfer experiments confirmed that higher force generation in TA1 group did not come at a cost of higher ATP consumption, as compared with Dob. Thus, our results suggest that increasing contractility by direct sarcomeric activation is less energetically costly than traditional inotropic approach via second messengers cAMP and calcium.

## Methods

The data that support the findings of this study are available from the corresponding author upon reasonable request.

### Experimental Animals

Forty-five 9-week-old male Sprague-Dawley rats weighing 280 to 320 g (Charles River Laboratories, Wilmington, MA) were housed in individual cages maintaining standard laboratory conditions and fed a regular pellet diet ad libitum conforming to the Guide for the Care and Use of Laboratory Animals published by the US National Institutes of Health (publication No. 8523, revised 1996) for 2 weeks before experiments. The protocol was approved by the Institutional Animal Care and Use Committee of the Boston University School of Medicine and Brigham and Women’s Hospital. The study was funded by Amgen.

### Development and Characterization of Inotropic Properties of Troponin Activator 1

**Figure 1.**
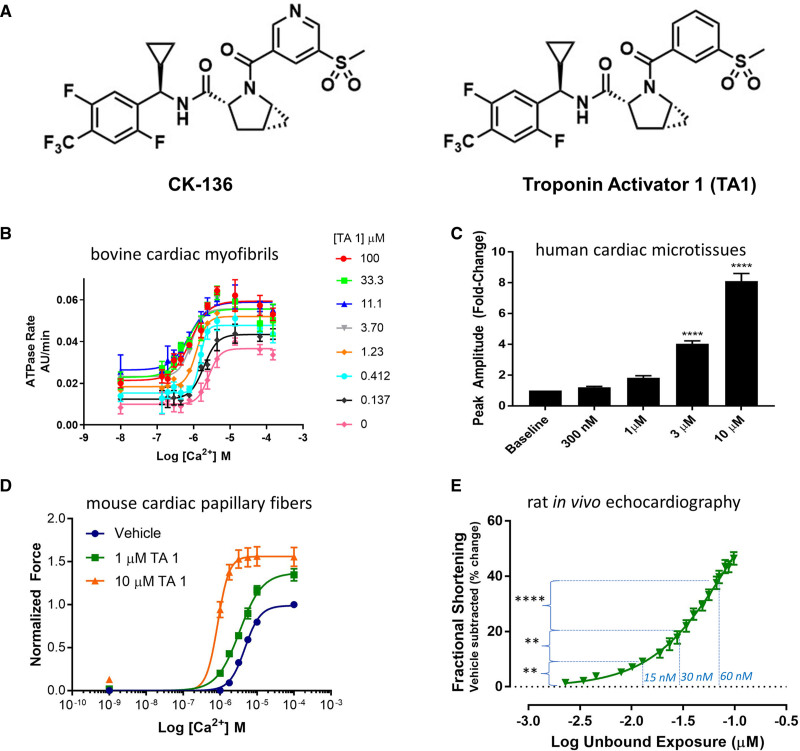
In vitro, ex vivo, and in vivo characterization of TA1. **A**, Structures of CK-136 (formerly known as AMG 594) and closely related analogue TA1 that was investigated in this study. **B**, TA1 sensitizes the ATPase activity of bovine cardiac myofibrils to activation by calcium. The ATPase activity of skinned bovine cardiac myofibrils was measured as described in Supplemental Methods. n=4. Fitted values are provided in Table S1. **C**, TA1 increases contractility of human cardiac microtissues. Maximum twitch amplitude of cardiac microtissues in response to increasing concentrations of TA1. Microtissues were treated with escalating doses, and data are normalized to each tissue’s baseline (ie, untreated) measurement. Summary data are mean±SEM. ****P<0.0001 (1-way ANOVA with Dunnett multiple comparisons test). n=6. **D**, TA1 increases calcium sensitivity and isometric tension at saturating calcium concentrations in permeabilized adult mouse cardiac muscle fibers. Force production by detergent-skinned adult mouse cardiac muscle fibers was measured at 10 °C. Data shown are mean±SEM. n=3 to 4. Fitted values are provided in Table S2. **E**, Effect of TA1 on fractional shortening (echocardiography; short axis, M mode) in the normal rat. Baseline-corrected, vehicle-subtracted fractional shortening was plotted against logarithmically transformed measured total whole blood concentration of TA1. The data are presented as mean±SEM. **P<0.01 and ****P<0.0001 (1-way ANOVA with Bonferroni multiple comparisons test). n=8 to 10. Vertical dotted lines show the effect of TA1 concentrations used in the nuclear magnetic resonance perfusion experiments 15 nmol/L TA1 (at −1.82 log), 30 nmol/L TA1 (−1.52 log), and 60 nmol/L TA1 (−1.22 log). AU indicates arbitrary units.

The structure of troponin activator (TA1) is identical to that of CK-136 (formerly known as AMG 594) with the noted exception of the replacement of the nitrogen in the 3-(methylsulfonyl)pyridine moiety of CK-136 with a carbon atom in TA1 (Figure 1A). Of note, we used higher TA1 concentrations in the in vitro assay, than those used in the perfused heart experiments, to see the maximum left shifting of the calcium dose-response curve. If the dose is pushed too high in vivo, or in isolated hearts, the heart stops beating because it remains in contraction. In vitro, however, we can escalate the dose to observe effects not seen in vivo. In vitro assays allowed us to see activation at saturating calcium concentrations. Full description of all Methods is in the Supplemental Material. Briefly:

#### ATPase Assay

Bovine cardiac myofibrils were prepared as per the Supplemental Material. Steady-state ATPase activity was measured using a pyruvate kinase and lactate dehydrogenase–coupled enzyme system. The reaction solution was freshly prepared and contained 4.0 mmol/L ATP, 1.6 mmol/L nicotinamide adenine dinucleotide, 3.0 mmol/L phosphoenolpyruvate, 30 unit/mL lactate dehydrogenase, and 30 unit/mL pyruvate kinase in assay buffer. All the working solutions were transferred to a 384-well black plate with clear bottom. The reaction was monitored at 340 nm at a 30-second interval for a total time of 1 hour.

#### Cardiac Microtissues

Cardiac microtissues were generated by TARA Biosystems, Inc (New York, NY), as described previously.^20,21^ Compound treatment was performed by TARA Biosystems, which was blinded to compound identity. To promote maturation of cardiac microtissues, custom chambers containing parallel carbon electrodes were used to provide electrical field stimulation using biphasic pulses of 2-ms duration, at twice the excitation threshold. Stimulation was started at 1 Hz and increased by 0.1 Hz increments daily to a maximum of 6 Hz. Matured microtissues were transferred to an environmentally controlled (37 °C, 5% CO_2_) test chamber, where tissues were equilibrated for 30 minutes at 1 Hz electrical stimulation. Measurements were taken after a 15-minute incubation with compound or vehicle. Video microscopy was then used to monitor deflection of poly(octamethylene maleate (anhydride) citrate) wires (100 fps with a ×10 objective in the blue channel). This procedure was then repeated for escalating compound dosages. Videos were then analyzed using a custom MATLAB (MathWorks, Inc, Natick, MA) algorithm to extract maximum twitch amplitude from raw force traces.

#### Skinned Papillary Fiber Force Mechanics

Papillary fibers were carefully dissected from the LVs of 12- to 14-week-old C57BL6 mice (n=4). Papillary fibers were skinned overnight and sequentially exposed to increasing calcium solutions at room temperature. For the measurement of maximal force and calcium sensitivity, a single fiber bundle was cycled through all calcium solutions 3×, first with vehicle (dimethyl sulfoxide) followed by increasing concentrations of TA1. Resulting curves were fit to a modified Hill Equation in GraphPad Prism.

#### Evaluation of TA1 in Normal Healthy Rats

Echocardiographic assessment was conducted in normal, healthy, isoflurane-anesthetized rats during an acute, continuous intravenous administration of TA1. Whole blood samples were collected from these same animals at prescribed intervals for analysis of TA1 exposures.

### Simultaneous Measurement of LV Contractile Function and HEP Concentrations by ^31^P NMR Spectroscopy

**Figure 2.**
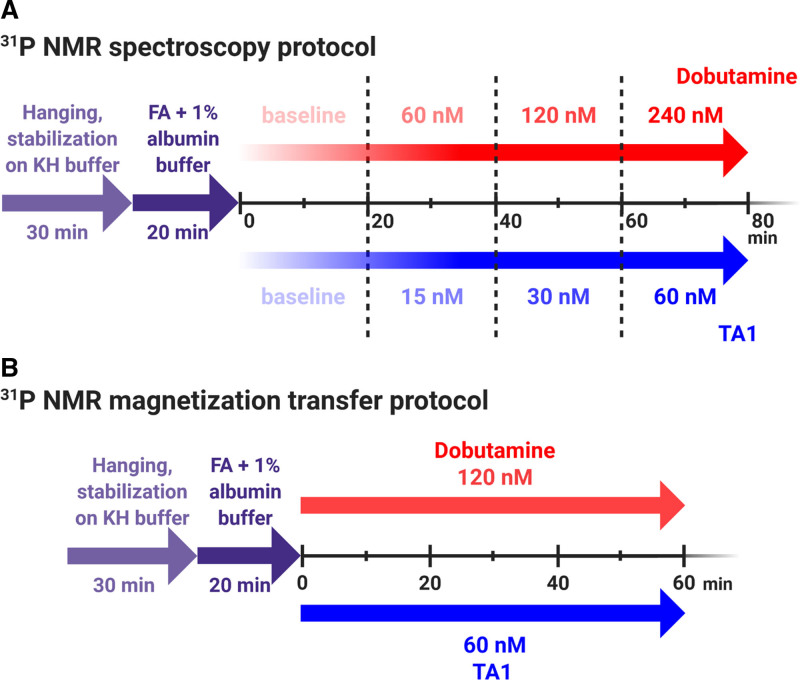
^31^P nuclear magnetic resonance (NMR) spectroscopy and magnetization transfer protocols. **A**, Two groups of isolated rat hearts were used to simultaneously measure left ventricular contractile function and high-energy phosphates by ^31^P NMR spectroscopy in a Langendorff heart preparation: (1) perfused with increasing concentrations of dobutamine (60, 120, and 240 nmol/L for 20 min each, n=11) and (2) perfused with increasing concentrations of TA1 (15, 30, and 60 nmol/L for 20 min each, n=15). **B**, Two separate sets of isolated rat hearts were used to measure contractile function, ATP synthesis, and high-energy phosphate flux by ^31^P NMR magnetization transfer technique in a Langendorff heart preparation. Given that 60 nmol/L TA1 and 120 nmol/L dobutamine increased the rate-pressure product to the similar extent in ^31^P NMR spectroscopy experiments (as per **A**), for magnetization transfer experiments, the hearts were (1) perfused with 120 nmol/L dobutamine (n=10) and (2) perfused with 60 nmol/L TA1 (n=9). FA indicates fatty acids; and KH buffer, Krebs-Henseleit buffer. Created with BioRender.com.

^31^P NMR spectroscopy was used to study high-energy phosphate (HEP) concentrations as per the study protocol (Figure 2A). In a separate cohort of hearts, magnetization transfer experiments were used to assess ATP synthesis rates (Figure 2B).

Please find detailed description of Methods in the Supplemental Material.

### Statistical Analysis

Results are presented as mean±SEM. One-way ANOVA with Dunnett multiple comparisons test was used to analyze the effect of incremental TA1 concentrations on the contractility of human cardiac microtissues; 1-way ANOVA with Bonferroni multiple comparisons test was used to analyze the effect of incremental TA1 concentrations on LV fractional shortening; averages of the 2 time point measurements per substance concentration were compared by paired *t* tests (baseline versus incremental concentrations of Dob or TA1) and unpaired *t* tests (60 nmol/L TA1 versus 120 nmol/L Dob) in LV contractile function and HEP data obtained by ^31^P NMR spectroscopy, and unpaired *t* tests (60 nmol/L TA1 versus 120 nmol/L Dob) were used to analyze the data form magnetization transfer experiments. Specific statistical tests and n per group are indicated in figure legends. All statistical analyses were performed using GraphPad Prism 7 (GraphPad Software, La Jolla, CA). *P*<0.05 was considered significant.

## Results

### Novel Myotrope TA1 Increases Cardiac Contractility In Vitro, Ex Vivo, and In Vivo

The structure of novel TA1 derived from a closely related analog troponin activator CK-136 (formerly known as AMG 594)^22^ is shown in Figure 1A. Note the only difference between these two troponin activators is a replacement of the nitrogen of the 3-(methylsulfonyl)pyridine moiety of CK-136 with a carbon atom in TA1. Troponin activators bind to troponin complex with binding thermodynamics recently defined by triple resonance (^1^H, ^13^C, ^15^N) structural NMR spectroscopy (L. Poppe et al, unpublished data, 2021; data available from the corresponding author upon request). The pharmacodynamic activity of TA1 in a series of in vitro, ex vivo, and in vivo assays (Figure 1B through 1E) closely mirrored that of CK-136. Cardiac myofibrils, isolated from LV bovine cardiac tissue, were used to measure ATPase activity under controlled free calcium concentrations. A left shift of the ATPase versus calcium curve was observed upon exposure to increasing concentrations of TA1 (Figure 1B; Table S1). TA1 was also tested in functionally matured cardiac microtissues comprised of human induced pluripotent stem cell–derived cardiomyocytes and human cardiac fibroblasts in a mechanically loaded systems.^20,21^ TA1 increased human microtissue contractility in a dose-dependent manner, with an 8.1-fold maximal increase in twitch amplitude relative to baseline (Figure 1C). To determine whether the potent in vitro effects of TA1 in cardiac myofibrils translate to ex vivo systems, freshly prepared detergent-skinned adult mouse cardiac fibers were evaluated. The pharmacodynamic effect of TA1 in cardiac-skinned muscle fibers in many respects mirrored that observed in isolated myofibrils (Figure 1D; Table S2). A left shift of the calcium dose-response curve, along with an increase in maximal tension, was observed upon exposing fibers to increasing concentrations of TA1. Next, the effect of TA1 in vivo was evaluated. Intravenous administration of TA1 in isoflurane-anesthetized normal rats resulted in a dose-dependent increase in fractional shortening, as measured by echocardiography (Figure 1E).

### TA1 Increases DevP Without Increasing HR or LVEDP

The effect of incremental Dob and TA1 concentrations on LV contractile function was compared with Dob-zero and TA1-zero baseline, respectively (protocol in Figure 2 and Table S3). In the second part of the study, the effect of 60 nmol/L TA1 was compared with 120 nmol/L Dob, as both drug concentrations increased the rate-pressure product (RPP) to a similar extent.

**Figure 3.**
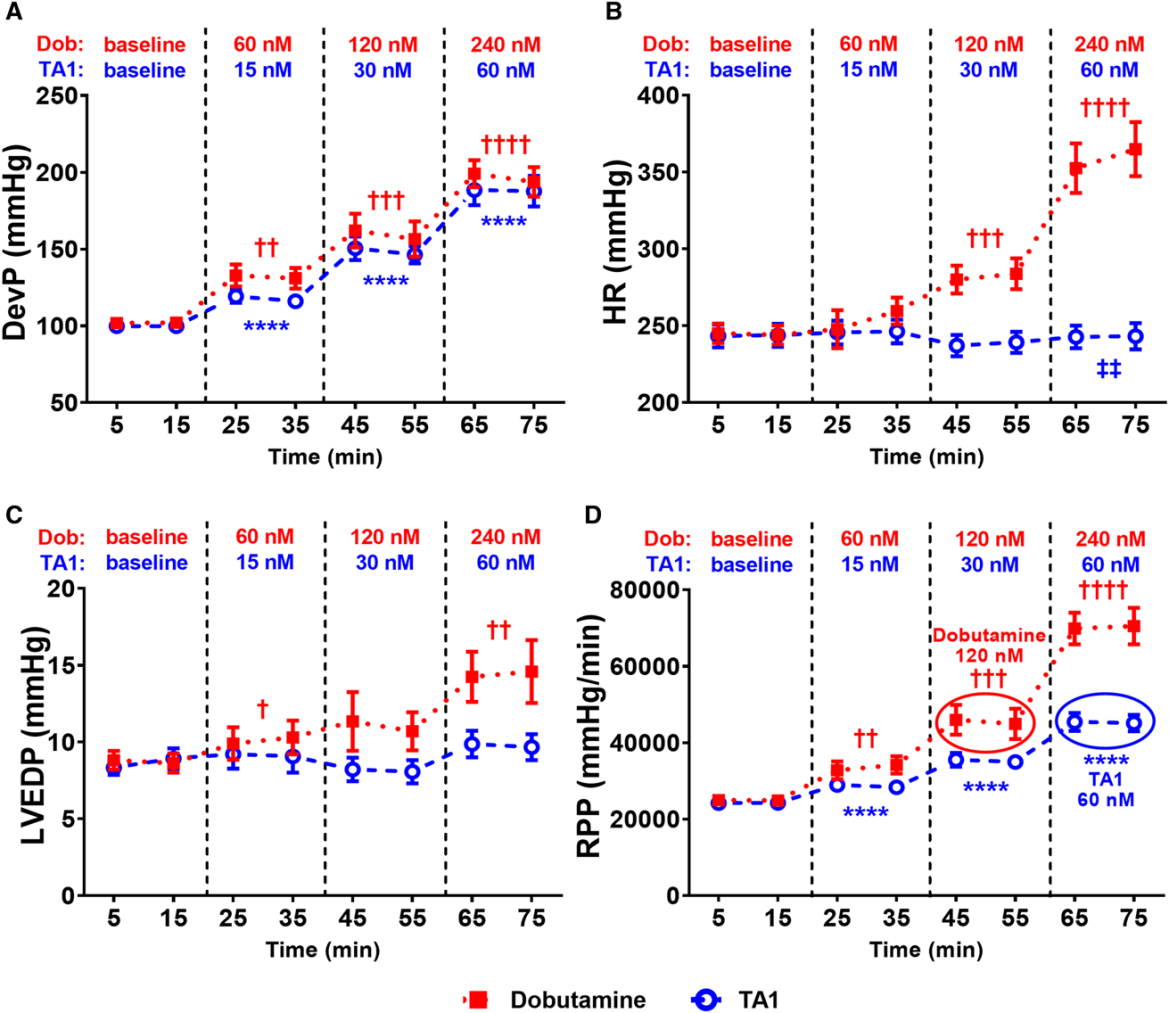
TA1 increases left ventricular developed pressure (DevP) and rate-pressure product (RPP) without affecting heart rate (HR) or left ventricular end diastolic pressure (LVEDP), as opposed to dobutamine (Dob). **A**, DevP increased progressively with increasing concentrations of both TA1 and Dob. **B** and **C**, HR (**B**) and LVEDP (**C**) underwent minor changes with TA1, yet both gradually increased with incremental concentrations of Dob. **D**, RPP increased progressively with increasing concentrations of both TA1 and Dob; 60 nmol/L TA1 increased RPP to the similar extent as 120 nmol/L Dob at lower HR (**B**). RPP was calculated as DevP×HR. As 60 nmol/L TA1 and 120 nmol/L Dob increased RPP to the similar extent, their effect is circled in **D**. n=15 (TA1) and 11 (Dob). Data shown are mean±SEM. Averages of the 2 time point measurements per substance concentration were compared. *P* values were obtained by paired *t* tests (baseline vs incremental concentrations of Dob or TA1) and unpaired *t* tests (60 nmol/L TA1 vs 120 nmol/L Dob). ****P<0.0001 vs TA1-zero baseline. †*P*<0.05, ††*P*<0.01, †††*P*<0.001, and ††††*P*=0.0001 vs Dob-zero baseline, respectively. ‡‡*P*<0.01 vs 120 nmol/L Dob. N for all time points is provided in Table S3.

LV systolic pressure (Figure S1A) and DevP (Figure 3A) increased progressively with increasing concentrations of Dob or TA1 in perfusate. Dob increased DevP by 29% (60 nmol/L Dob; *P*<0.01), 56% (120 nmol/L Dob; *P*<0.001), and 95% (240 nmol/L Dob; *P*<0.0001) versus baseline; TA1 increased DevP by 18% (15 nmol/L TA1; *P*<0.0001), 49% (30 nmol/L TA1; *P*<0.0001), and 89% (60 nmol/L TA1; *P*<0.0001) versus baseline (Figure 3A). LVEDP remained unchanged with TA1 (*P*=0.2596, *P*=0.7031, and *P*=0.1790), yet it gradually increased with incremental concentrations of Dob (by 16%, 26%, and 66% versus baseline; *P*<0.05, *P*=0.0714, and *P*<0.01, respectively; Figure 3C). HR markedly increased with Dob increments (by 4%, 15%, and 45% versus baseline; *P*=0.1298, *P*<0.001, and *P*<0.0001, respectively) and remained unaffected by TA1 throughout the protocol (Figure 3B). The RPP progressively increased with increasing concentrations of Dob (by 34%, 82%, and 181% versus baseline; *P*<0.01, *P*<0.001, and *P*<0.0001, respectively) and TA1 (by 18%, 46%, and 87% versus baseline; *P*<0.0001 for all; Figure 3D).

Since 60 nmol/L TA1 increased RPP to the similar extent as 120 nmol/L Dob (45 118±2163 versus 44 896±3983 mm Hg/min), we compared the effect of both drugs on LV contractile function: TA1 achieved 18% higher DevP (*P*=0.0651) at 14% lower HR (*P*<0.01) and 11% lower LVEDP (*P*=0.4383) compared with Dob (Figure 3). Thus, equivalent concentration of TA1 increases DevP more than Dob, without increasing HR or worsening of the end diastolic pressures.

### HEPs and Free Energy of ATP Hydrolysis (ΔG_~ATP_) Were Preserved by TA1 but Depleted by Dob

**Figure 4.**
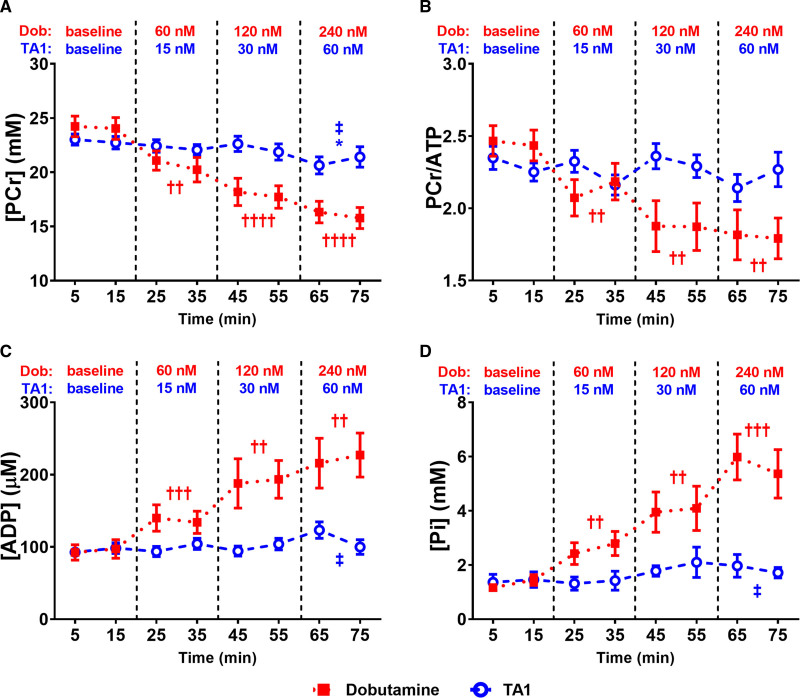
As opposed to dobutamine (Dob), TA1 preserves high-energy phosphates during high workload. Phosphocreatine (PCr; **A**) and PCr/ATP (**B**) progressively declined with increasing Dob concentrations and remained grossly unaffected by TA1. Calculated cytosolic free (ADP; **C**) and inorganic phosphate (Pi; **D**) progressively increased with Dob increments and were preserved by TA1. n=15 (TA1) and 11 (Dob). Data shown are mean±SEM. Averages of the 2 time point measurements per substance concentration were compared. *P* values were obtained by paired *t* tests (baseline vs incremental concentrations of Dob or TA1) and unpaired *t* tests (60 nmol/L TA1 vs 120 nmol/L Dob). *P<0.05 vs TA1-zero baseline. ††*P*<0.01, †††*P*<0.001, and ††††*P*=0.0001 vs Dob-zero baseline, respectively. ‡*P*<0.05 vs 120 nmol/L Dob. N for all time points is provided in Table S3.

Simultaneously with assessing LV contractile function, myocardial HEPs were measured by ^31^P NMR spectroscopy (Figure 2). The ATP remained unchanged throughout the protocol and was similar in both groups (Figure S1B). PCr significantly decreased with Dob increments (by 14%, 26%, and 34% versus baseline; *P*<0.01, *P*<0.0001, and *P*<0.0001, respectively) and was minimally affected by TA1 (*P*=0.1852, *P*=0.4848, and *P*<0.05, −8% versus baseline) throughout the protocol (Figure 4A). Similarly, PCr/ATP progressively declined with increasing Dob concentrations (by 13%, 23%, and 27% versus baseline; *P*<0.01 for all) and TA1 had no significant effect (*P*=0.6074, *P*=0.6624, and *P*=0.4066; Figure 4B). Neither Dob nor TA1 affected total creatinine or intracellular pH (Figure S1C and S1D). Both inorganic phosphate (Pi) and the cytosolic free ADP progressively increased with Dob increments by 101%, 209%, 336% (*P*<0.01, *P*<0.01, *P*<0.001) and 45%, 101%, 134% (*P*<0.001, *P*<0.01, *P*<0.01), respectively, yet during TA1 treatment, Pi (*P*=0.5052, *P*=0.2966, and *P*=0.1282) and ADP (*P*=0.4425, *P*=0.8893, and *P*=0.0788) remained without significant change (Figure 4C and 4D).

**Figure 5.**
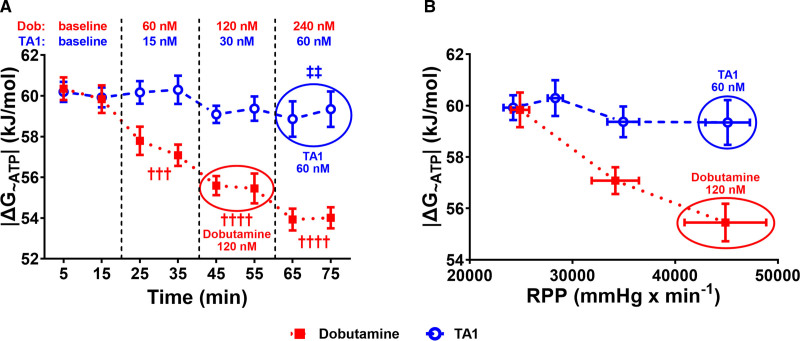
TA1 preserves while dobutamine (Dob) depletes the free energy of ATP hydrolysis (|ΔG_~ATP_|). **A**, Absolute value |ΔG_~ATP_| progressively declined with Dob increments and was preserved by TA1. **B**, Plotting |ΔG_~ATP_| against rate-pressure product (RPP) expresses the combined effect of TA1 or Dob on cardiac energetics and contractile function. TA1 preserved |ΔG_~ATP_| (**A**) and maintained the |ΔG_~ATP_|/RPP relationship horizontal compared with 120 nmol/L Dob (**B**) that caused decline in |ΔG_~ATP_|/RPP. Thus, TA1 achieved similar RPP at preserved |ΔG_~ATP_|. As 60 nmol/L TA1 and 120 nmol/L Dob increased RPP to the similar extent at different |ΔG_~ATP_|, their effects are circled in both **A** and **B**. In **B**, the Dob concentrations from **left** to **right** are Dob-zero baseline, 60 nmol/L, and 120 nmol/L, and the TA1 concentrations from **left** to **right** are TA1-zero baseline, 15 nmol/L, 30 nmol/L, and 60 nmol/L. n=15 (TA1) and 11 (Dob). Data shown are mean±SEM. Averages of the 2 time point measurements per substance concentration were compared. *P* values were obtained by paired *t* tests (baseline vs incremental concentrations of Dob or TA1) and unpaired *t* tests (60 nmol/L TA1 vs 120 nmol/L Dob). †††*P*<0.001 and ††††*P*=0.0001 vs Dob-zero baseline, respectively. ‡‡*P*<0.01 vs 120 nmol/L Dob. N for all time points is provided in Table S3.

Since 60 nmol/L TA1 increased RPP to a similar extent as 120 nmol/L Dob (Figure 3D), we compared effects of these concentrations on HEP: PCr was higher by 16% (*P*<0.05), Pi was lower by 50% (*P*<0.05), and ADP lower by 39% (*P*<0.05) in the TA1 group (Figure 4). In line with lower ADP, 60 nmol/L TA1 maintained higher |ΔG_~ATP_| compared with 120 nmol/L Dob (*P*<0.01; Figure 5).

|ΔG_~ATP_| markedly decreased with Dob increments from 60.1±0.62 kJ/mol at baseline to 57.44±0.61 kJ/mol (at 60 nmol/L Dob, *P*<0.001), 55.52±0.6 kJ/mol (at 120 nmol/L Dob, *P*<0.0001), and 53.97±0.53 kJ/mol (at 240 nmol/L Dob, *P*<0.0001), yet it remained without significant change by TA1 (*P*=0.4013, *P*=0.2463, and *P*=0.0877; Figure 5A). Plotting |ΔG_~ATP_| against RPP reveals the effects of Dob and TA1 on the relationship between cardiac energetics and contractile function. Indeed, the |ΔG_~ATP_|/RPP relationship declines with Dob increments, yet no such trend was detected in TA1 perfused hearts (Figure 5B).

### Despite Higher DevP and Wall Stress, TA1 Has a Neutral Effect on Mitochondrial ATP Synthesis Rate

**Figure 6.**
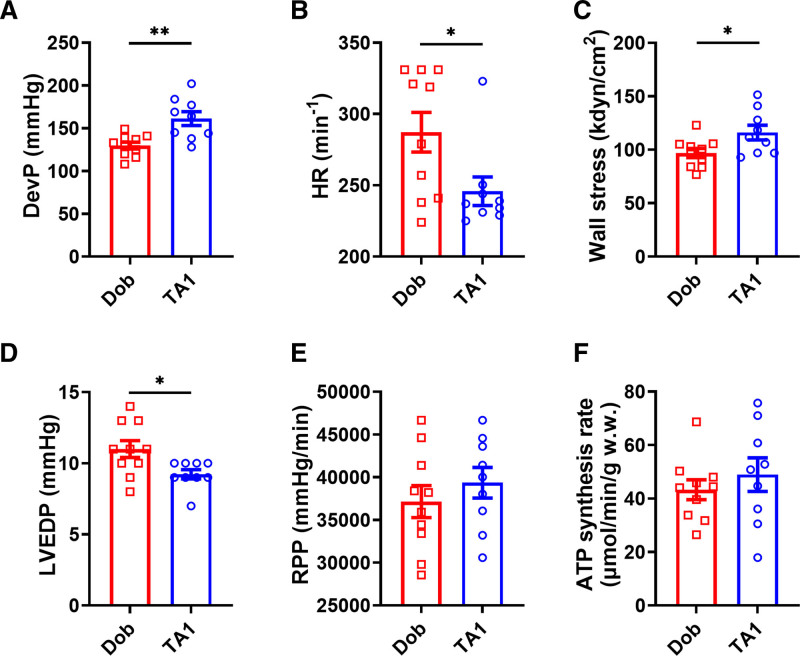
TA1 at 60 nmol/L achieves similar rate-pressure product (RPP) at higher developed pressure (DevP), wall stress and lower heart rate (HR), and left ventricular end diastolic pressure (LVEDP) compared with 120 nmol/L dobutamine (Dob), with neutral effect on ATP synthesis rate. **A** through **E**, During magnetization transfer, 60 nmol/L TA1 achieved higher DevP and wall stress (**A** and **C**), lower LVEDP (**D**) and HR (**B**), and similar RPP (**E**) compared with 120 nmol/L Dob. ATP synthesis rate was similar between the groups. **F**, n=9 (in 60 nmol/L TA1 group) and 10 (in 120 nmol/L Dob group). Data shown are mean±SEM. *P* values were obtained by unpaired *t* tests. *P<0.05 and **P<0.01, respectively. w.w. indicates wet weight.

As 60 nmol/L TA1 and 120 nmol/L Dob increased RPP to the similar extent in previous experiments, we compared the effect of these substance concentrations on contractile function and ATP synthesis rate using a 31P NMR magnetization transfer technique in a separate cohort of hearts. During the magnetization transfer protocol, 60 nmol/L TA1 and 120 nmol/L Dob recapitulated their effect on LV contractile function measured in previous experiments: 60 nmol/L TA1 achieved similar RPP (39 351±1790 mm Hg/min versus 37 141±1879 mm Hg/min; *P*=0.4088

), higher DevP (by 24%; *P*<0.01), and lower LVEDP and HR (by 16% and 14%, respectively; *P*<0.05 for both) compared with 120 nmol/L Dob (Figure 6A, 6B, 6D, and 6E; Figure S2A). Interestingly, 60 nmol/L TA1 increased LV systolic wall stress by 20% more (*P*<0.05) compared with 120 nmol/L Dob (Figure 6C). There was no significant difference in ATP synthesis rate (48.94±6.29 versus 43.3±3.72 µmol/min/g; *P*=0.4392) between the groups (Figure 6F; Figure S2B and S2C).

## Discussion

In this study, we showed that novel myotrope TA1 increased contractility in vitro, ex vivo, and in vivo. Unlike Dob, TA1 increased RPP through increasing DevP without altering the HR, HEPs, or LVEDP. Dob depleted PCr, increased ADP, and decreased free energy of ATP hydrolysis (ΔG_~ATP_) and worsened diastolic filling pressures. Thus, as opposed to a traditional inotrope, TA1 uniquely increased myocardial contractility without disturbing the delicate balance between ATP supply and demand.

### Failing Heart Is Energy Starved

Despite advances in treatment, the epidemiology of HF remains alarming as it afflicts 6.5 million Americans,^4,23^ while HF prevalence continues to rise.^24^ From a physiological perspective, a complex of compensatory mechanisms, including sympathetic tone and neurohumoral pathways, is activated to preserve perfusion of peripheral tissues in systolic HF. However, chronic exposure to this neurohumoral activation leads to increased myocardial oxygen consumption, cell death, and pathological remodeling.^11,23,24^ As a result, ATP demand outstrips ATP supply, PCr falls, while ATP may remain constant, but the free ADP and Pi rise. The increased ADP affects activity of cellular ATPases such as SERCA (sarcoplasmic reticulum calcium ATPase), thus limiting contractility even at unchanged ATP levels, while ATP concentration decreases only in end-stage HF.^1,9,25^ Indeed, the cardiac energy status as expressed by the PCr/ATP ratio is reduced in HF and correlates with systolic and diastolic cardiac function and predicts mortality in HF better than LV ejection fraction and New York Heart Association class.^9,25^ The free energy of ATP hydrolysis (ΔG_~ATP_) is even more accurate measure of myocardial energetic reserve and describes the chemical potential in a molecule of ATP to perform work and represents the driving force of all ATP-consuming processes.^25–29^ Once ΔG_~ATP_ is decreased, regardless of the cause, the heart has reduced contractile reserve and is at risk for acute mechanical failure during challenges such as an abrupt increase in work demand, hypoxia, ischemia, or arrhythmia.^25,27^ Importantly, currently approved HF therapies improve myocardial energy balance by blocking neurohumoral activation—an effect likely contributing to their improved prognosis.^9,25,30^

### Addition of Myotropes Could Significantly Augment HF Medical Therapy

Targeting of neurohumoral activation by β-blockers and inhibitors of renin-angiotensin-aldosterone system decreases myocardial oxygen consumption and improves long-term outcomes in chronic HF with reduced ejection fraction.^1,9^ These therapies decrease afterload and maintain cardiac output via peripheral vasodilation and decrease in systolic blood pressure. Such unloading of the heart leads to improved clinical outcomes, even without addressing the central driver of HF with reduced ejection fraction pathogenesis—the impaired cardiac contractility.^11,23^ In everyday clinical practice, however, low blood pressure frequently limits the use of optimal doses of neurohumoral inhibitors in HF management.^3,31^ In fact, registry studies show that only 20% of HF patients receive target doses of guideline-directed medical therapy,^32^ and real-word HF survival is much worse than in clinical trials.^33^ Given its known negative impact on long-term survival, chronic inotropic support is only used in advanced and end-stage HF to improve quality of life or as a bridge to heart transplant.^3,23,34^ Thus, if myotropes were shown to be free of the negative impact on survival, they could have much broader indications than currently available inotropes. Moreover, addition of myotropes to the standard therapy could facilitate optimization of vasodilating and diuretic agents, titration of which is otherwise frequently limited by patients’ low blood pressure.^3,31^

### Myotropes May Increase Contractility While Preserving Cardiac Energetics

The increased myocardial oxygen consumption is a common denominator of increased cardiac contractility.^35^ However, traditional inotropes (ie, calcitropes) tend to additionally impair energy efficiency with resulting oxygen wasting effect^36^ and to associate with adverse prognosis in HF,^2^ thus supporting an almost century-old hypothesis that the failing heart is energy starved.^9,25,35^

In this study, we hypothesized that direct activation of the cardiac sarcomere would improve cardiac performance without the adverse energetic effects of traditional inotropes. Conceptually, the cardiac sarcomere can be activated either by directly activating the cardiac myosin^11,37,38^ or by sensitizing the regulatory proteins to calcium.^11,12^ The first approach, direct stimulation of myosin with omecamtiv mecarbil (OM), has recently been shown to improve HF outcomes in the GALACTIC-HF clinical trial (Global Approach to Lowering Adverse Cardiac Outcomes Through Improving Contractility in Heart Failure).^10^ OM binds to the catalytic domain of cardiac myosin and increases the number of myosin heads that enter the force-producing state, and it also appears to decrease inefficient actin-independent noncontractile energy usage. Importantly, OM does not augment calcium-dependent second messenger signaling to increase contractile function.^11,12^ CK-136 (formerly known as AMG 594) has been developed to test the second, calcium-sensitizing approach. CK-136 is first-in-class, small-molecule selective cardiac troponin activator shown to selectively sensitize the cardiac troponin-tropomyosin regulatory complex to calcium (A.S. Motani et al, unpublished data, 2021; data available from the corresponding author upon request). Thus, in the same manner as OM, CK-136 should increase cardiac contractility with no effect on calcium transients. In this study, we use TA1—a novel molecule developed by replacement of the 3-(methylsulfonyl)pyridine moiety of CK-136 with a carbon atom (Figure 1A). Similarly to CK-136, TA1 dose dependently increases measures of contractility, LV fractional shortening, and ejection fraction. In isolated hearts, TA1 enhanced contractile performance without affecting HR or LVEDP, while simultaneously preserving HEPs and ΔG_~ATP_. In contrast, Dob increased LV contractility with profound HR and LVEDP elevation and simultaneously depleted HEP and decreased ΔG_~ATP_, indicative of acute ATP demand/supply mismatch often seen in acute HF. The energetic depletion to Dob seen in our study is consistent with prior reports of worsening energetics during inotropic stimulation with traditional calcitropes.^15,39–41^ To further assess this energy-sparing effect, we studied the effect of moderate dose of TA1 on mitochondrial ATP synthesis in a separate set of experiments. TA1 achieved similar estimated work at higher DevP and lower HR and LVEDP compared with Dob, while no intergroup differences were found in mitochondrial ATP synthesis. Collectively, these data suggest more efficient energy utilization for contraction in TA1-treated hearts.

### Determinants of Improved Energy Balance During TA1-Induced High Workload

Traditional inotropes increase contractility associated with exaggerated myocardial oxygen consumption^42–44^ and frequently lead to worsened energy balance reflected by decrease in PCr/ATP and ΔG_~ATP_.^39–41^ The potential benefit of novel myotropes is preservation of energy balance so the conserved energy may then be redirected for cellular repair and promotion of mitochondrial health with reduction of oxidative stress. Indeed, myotropes do not appear to drive oxygen consumption like more conventional agents.^8^ This is likely due to lower energy cost of calcium cycling^8^ and lack of chronotropic response.^36,45^ Notably, in our experiments, HR dose dependently increased in the Dob group while remaining unchanged in the TA1 group (Figure 3B). Elevated LVEDP and diastolic wall tension increase energy expenditure to the similar level as systolic force generation.^29,46^ Thus, our data suggest that elevated LVEDP by Dob may further contribute to increased myocardial energy demand. In contrast, TA1 increases cardiac work by increasing DevP with preserved HR and LVEDP likely contributing to its myocardial energy-sparing effect. Additionally, in clinical realm, lower LVEDP may directly translate to lower pulmonary pressures and improved symptoms of shortness of breath.^35^

### Benefit of Myotropes May Be More Pronounced in More Advanced HF

**Figure 7.**
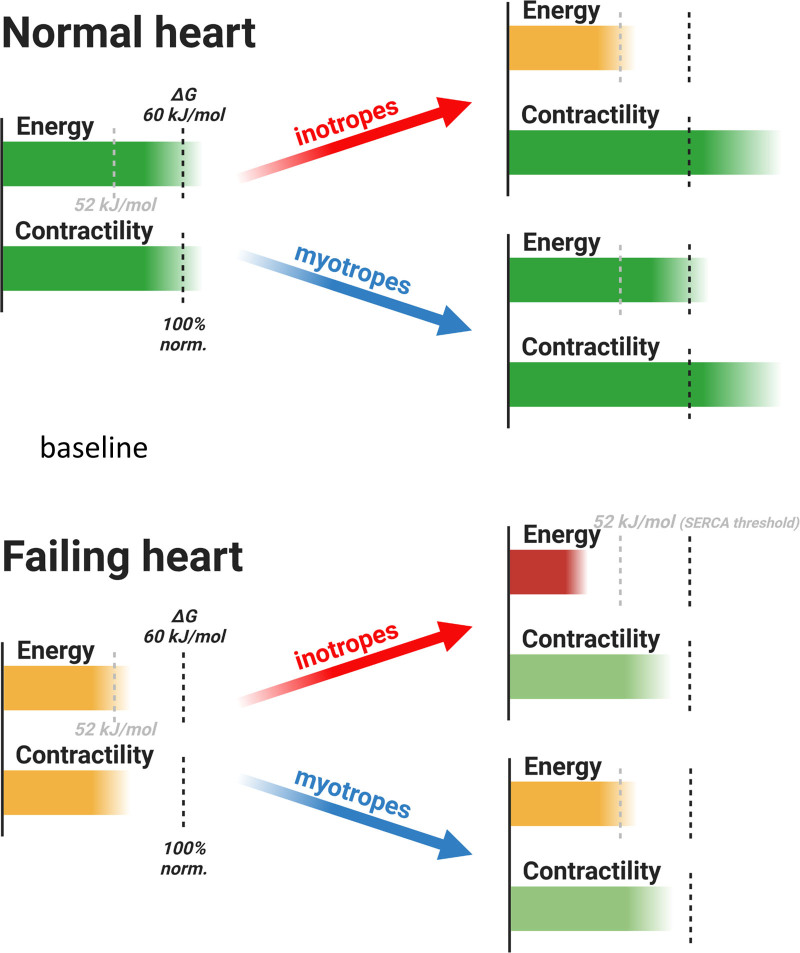
Inotropes vs myotropes in heart failure (HF). During inotrope infusion in the normal heart, |ΔG_~ATP_| decreases but stays above the value of 52 kJ/mol—a critical value for normal SERCA (sarcoplasmic reticulum calcium ATPase) function. SERCA is a major regulator of calcium homeostasis and contractility in the heart, and thus SERCA dysfunction may contribute to both diastolic dysfunction and loss of contractile reserve with increased work demand. In contrast, when inotropes are used in HF, myocardium starts at lower level of |ΔG_~ATP_|; any further decrease will likely cross this threshold with resulting SERCA dysfunction and diastolic and systolic dysfunction. Given their energy-sparing properties, myotropes may increase contractility with no further worsening in ATP supply/demand mismatch. Created with BioRender.com.

The initial insult in HF with reduced ejection fraction is related to loss of contractility due to sarcomeric malfunction or tissue loss from ischemia or inflammation. Mitochondrial function is generally unaffected in initial stages and energetics progressively worsens as LV dilation and HF progress.^9,25^ Thus, while our study showed energetic benefit of TA1 in normal rat hearts, it is tempting to speculate that with more severely impaired energetics, the beneficial energy-sparing effect of myotropes will be even more pronounced. Indeed, the results of the GALACTIC-HF trial suggest that patients with more progressed HF (subgroup of patients with median LV ejection fraction ≤28%,) derive 2-fold higher benefit from myotropic therapy with OM than the broad patient population (hazard ratio, 0.84 versus 0.92).^10^ Such clinical outcomes are consistent with our experimental results suggesting that the energy-sparing effect of TA1 could be magnified in energetically deprived myocardium (Figure 7). As a major regulator of calcium homeostasis and contractility, SERCA is exquisitely sensitive to energy deprivation.^15,27,47^ Thus SERCA dysfunction contributes to both diastolic dysfunction and loss of contractile reserve with decreased |ΔG_~ATP_|.^15,16^ Notably, Dob infusion in our study decreased ΔG_~ATP_ to values that stayed above 52 kJ/mol—a critical value for normal SERCA function.^15,16,27,47^ In contrast to normal hearts used in our study, failing myocardium starts at lower level of |ΔG_~ATP_|, and any further decrease in ΔG_~ATP_ by inotropes would likely cross the SERCA threshold with further energetic and contractile deterioration. Thus, our results suggest that, in contrast to inotropes, myotropes have the potential to preserve myocardial energetics and thus increase contractility with minimal negative impact on calcium cycling, diastolic function, or contractile reserve in a failing heart (Figure 7).

### Limitations

Our experiments were performed on otherwise healthy hearts from animals without any cardiovascular condition. This limits the generalizability of the current findings but opens avenues for further investigation (Figure 7). Moreover, our assessment of the effect of TA1 on diastolic function is limited by the large difference in HR between the TA1 and Dob groups. The diastolic dysfunction is known to occur before systolic dysfunction with acute energy depletion: The acute fall in the free energy in ATP hydrolysis |ΔG_~ATP_| results in slowing of SERCA, as well as cross-bridge cycling.^27^ While we^16,18,19^ and others^48^ reported the correlation between worsened energetics and diastolic dysfunction in both mouse models and patients, no definite conclusions can be drawn about diastolic function benefit by TA1 in our study. To properly compare the effect of myotropes on diastolic function, there should be a mechanism to control for HR differences between the studied groups.

Reproduction of our findings in clinical investigation is limited by availability of cutting-edge technology to measure |ΔG_~ATP_| in patients.^49^ The ^31^P NMR signal from 2,3-diphosphoglycerate in red blood cells interferes with the assessment of chemical shift (indicative of intracellular pH) and concentration of Pi in vivo. These values, along with the total creatine concentration that we measure from frozen tissue by high-performance liquid chromatography, are needed for the calculation of |ΔG_~ATP_|. However, clinical assessment of PCr/ATP is feasible. While the PCr/ATP ratio does not directly measure the thermodynamic driving force for ATP-dependent processes, it provides a useful assessment of myocardial energetics in patients.^9,48^

### Conclusions

In summary, our findings demonstrate that novel myotrope TA1 increases contractility while preserving the ATP supply/demand balance.

This observation provides basis for further investigation of myotropic treatment as a therapeutic strategy for HF with reduced ejection fraction characterized by decreased contractility and worsened energetics. Moreover, myotropes may prove particularly helpful in advanced disease when patient’s low blood pressure limits optimal titration of current guideline-directed medical therapy.

## Article Information

### Sources of Funding

This study was sponsored by Amgen. Dr Luptak was also supported by American Heart Association Fellow-to-Faculty Award 15FTF25890062.

### Disclosures

Drs Motani and Reagan are Amgen employees and shareholders; K.K. Nguyen and Drs Liu, Rock, Wang, Hale, and Karamanlidis are Amgen employees; Dr Slater was an Amgen employee at the time of the study; and Drs Hartman and Malik are Cytokinetics employees and shareholders. The other authors report no conflicts.

### Supplemental Material

Supplemental Methods

Tables S1–S3

Figures S1 and S2

## Supplementary Material


