# Supplementary material for: Novel Small-Molecule Troponin Activator Increases Cardiac Contractile Function Without Negative Impact on Energetics
Source: Circ Heart Fail. 2021 Nov 8;15(3):e009195. doi: 10.1161/CIRCHEARTFAILURE.121.009195 (PMC8920024; doi:10.1161/CIRCHEARTFAILURE.121.009195)

SUPPLEMENTAL MATERIAL

Supplemental Methods

Preparation of cardiac myofibrils - 250 g to 300 g of bovine heart left ventricle tissue was coarsely chopped in a Waring@ blender with cold buffer A (25 mM PIPES, 50 mM KCl, 5.0 mM $MgCl_2$, 1.0 mM NaN_3, 1.0 mM DTT, pH 7.0) and then homogenized with 1300D Polytron™ Homogenizer (12 mm generator) for 3 x 2 min at 30,000 rpm on ice. The homogenate was centrifuged at 5,000 rpm (Sorvall RC 6 Plus with F10S-6X500Y Rotor) for 15 min. After centrifugation, all the supernatant was discarded and a light brown layer of mitochondria, sarcolemma and sarcoplasmic reticulum on the top of pellet was scrapped off. The pellet was homogenized with buffer B (25 mM PIPES, 50 mM KCl, 5.0 mM $MgCl_2$, 1.0 mM NaN_3, 1.0 mM DTT, 2 mM EGTA, pH 7.0). The suspension was then centrifuged at 9,000 rpm (Sorvall RC 6 Plus with F10S-6X500Y Rotor) for 15 min. The supernatant was discarded and the light brown layer on top of pellet was scrapped off. The pellet was homogenized with buffer C containing 1% triton X-100 (25 mM PIPES, 50 mM KCl, 5.0 mM $MgCl_2$, 1.0 mM NaN_3, 1.0 mM DTT, 2 mM EGTA, 1% triton X-100, pH 7.0) to remove mitochondrial, sarcolemmal and sarcoplasmic reticular membranes. After the pellet was fully resuspended in buffer C, the suspension was then centrifuged at 4,200 rpm (Sorvall RC 6 Plus with F10S-6X500Y Rotor) for 10 min. This sequence of resuspension, homogenization and centrifugation was repeated for another two times. The resulting light brown pellet was filtered through Nitex nylon mesh 630 MICRON (Genesee Scientific Corp catalogue No. NC9432449) to remove any residue coarse tissue. Then the previous sequence of resuspension, homogenization and centrifugation was repeated for another two times. Finally, the triton X-100 was removed with buffer A wash for three times. In each wash, the pellet was homogenized in Buffer A, and the

suspension was centrifuged at 4,200 rpm (Sorvall RC 6 Plus with F10S-6X500Y Rotor) for 10 min; all the supernatant was discarded and the top light brown layer of each pellet was removed. After three washes, the pellet was homogenized in buffer A. The homogenate obtained after three wash was white in color. The homogenate was then aliquoted and stored at -80°C.

ATPase assay - Steady-state ATPase activity was measured using a pyruvate kinase and lactate dehydrogenase–coupled enzyme system. The assay was performed in assay buffer containing 15 mM PIPES (pH 7.0), 5.0 mM $MgCl_2$, 10 mM KCl, 1.0 mM NaN_3 and 1.0 mM freshly added DTT. The reaction solution was freshly prepared and contained 4.0 mM ATP, 1.6 mM NADH, 3.0 mM phosphoenolpyruvate, 30 unit/ml lactate dehydrogenase and 30 unit/ml pyruvate kinase in assay buffer. All the working solutions were transferred to a 384-well black plate with clear bottom. The reaction was monitored at 340 nm at 30 second interval for a total time of one hour.

Cardiac microtissues Cardiac microtissues were generated by TARA Biosystems Inc. (New York, NY, USA) as previously described.[20,21] Compound treatment was carried out by TARA Biosystems, who were blinded to compound identity. To promote maturation of cardiac microtissues, custom chambers containing parallel carbon electrodes were used to provide electrical field stimulation using biphasic pulses of 2 ms duration, at twice the excitation threshold. Stimulation was started at 1 Hz and increased by 0.1 Hz increments daily to a maximum of 6 Hz. Matured microtissues were transferred to an environmentally controlled (37ºC, 5% CO_2) test chamber, where tissues were equilibrated for 30 minutes at 1Hz electrical stimulation. Tissues were pre-conditioned to compound addition by media extraction and injection in the test chamber twice at 10-minute intervals.

Compound was then added and 1/3 of media in the test chamber was gently pipetted in the test chamber to mix. Measurements were taken after a 15-minute incubation with compound or vehicle. Video microscopy was then used to monitor deflection of poly(octamethylene maleate (anhydride) citrate) (POMaC) wires; images of autofluorescent POMaC wires were acquired at 100 fps with a 10x objective in the blue channel (λex = 350 nm, λem = 470 nm). This procedure was then repeated for escalating compound dosages. Videos were then analyzed using a custom MATLAB (MathWorks, Inc., Natick, MA, USA) algorithm to extract maximum twitch amplitude from raw force traces.

Skinned papillary fiber force mechanics - Papillary fibers were carefully dissected from the left ventricles of 12-14 weeks old C57BL6 mice (n=4). Papillary fibers were skinned over night at 4 ^{0}C in 1% Triton X-100 in relaxing buffer [55.74 mM potassium propionate, 7 mM ethylene glycol bis(2-aminoethyl)tetraacetic acid, 100 mM N,N-bis(2-hydroxyethyl)-2-amino ethanesulfonic acid, 0.02 mM $CaCl_2$, 5.5 mM $MgCl_2$, 5 mM dithithreitol, 15 mM creatine phosphate and 4.7 mM ATP. Skinned papillary fibers were sequentially exposed to increasing calcium solutions made by mixing relaxing buffer and activating solution [relaxing buffer with the addition of 7 mM $CaCl_2$] at room temperature. For the measurement of maximal force and calcium sensitivity, a single fiber bundle was cycled through all calcium solutions three times, first with vehicle (DMSO) followed by increasing concentrations of TA1. Resulting curves where fit to a modified Hill Equation in GraphPad Prism.

Evaluation of TA1 in normal healthy rats - ***in vivo rodent pharmacodynamics***

All animal care and use was in compliance with the Amgen IACUC protocol, appropriate guidelines of the test facility, and animal welfare regulations. Normal, healthy, male CD rats aged 9-10 weeks, were obtained from Charles River, Hollister, CA, USA. On the day of study, rats were implanted with a jugular vein catheter under isoflurane anesthesia for intravenous (IV) test article delivery. Infusion of vehicle or a fixed concentration of TA1 was then initiated with the aid of a programmable syringe pump. The dose delivered was escalated by increasing the infusion rate in a stepwise manner at 15-minute intervals. Time-matched controls were generated by dosing rats designated for the "vehicle" group with vehicle only. Samples of whole blood (5uL; expressed from a small nick in the tail) were collected at 5 min intervals for subsequent bioanalytical determination of test article exposure. In this manner both pharmacodynamics (PD; echo measures) and pharmacokinetic (PK; exposure) were determined in the same animal.

An ultrasound probe (Vevo2100) was positioned to acquire M-Mode images at the midventricular level as indicated by the presence of papillary muscles on the short axis view and fractional shortening (FS) was acquired. Baseline-corrected, vehicle-subtracted FS was plotted against logarithmically-transformed measured total whole blood concentrations of TA1. A 4-parameter nonlinear fit (bottom constrained to zero, variable slope; GraphPad Prism) was performed.

Simultaneous measurement of LV contractile function and high-energy phosphate concentrations (HEP) by ^{31}P NMR spectroscopy

Isolated hearts of twenty-six rats were used to simultaneously measure LV contractile function and HEP by ^{31}P NMR spectroscopy. There were two groups of hearts: i) perfused with increasing concentrations of dobutamine (60 nM, 120 nM, and 240 nM for 20 min

each, n=11), and ii) perfused with increasing concentrations of TA1 (15 nM, 30 nM, and 60 nM for 20 min each, n=15) (Figure 2A). Left ventricular contractile function was assessed in an isolated retrograde-perfused Langendorff heart preparation as described previously.[16-18] Rats were heparinized (4U IP per g of body weight) and anesthetized with isoflurane. The heart was excised and perfused at a constant pressure of 80 mm Hg. The perfusate (modified Krebs-Henseleit buffer) consisted of the following (in mmol/L): NaCl 118, $NaHCO_3$ 25, KCl 5.3, $CaCl_2$ 2, $MgSO_4$ 1.2, EDTA 0.5, glucose 10, palmitate 0.4 bound to 1% albumin equilibrated with 95% O_2 and 5% CO_2 (pH 7.4). Dobutamine and TA1 were added to the perfusate in incremental concentrations as per study protocol (Figure 2).

To assess left ventricular (LV) contractile function, a water-filled balloon was inserted into the LV. After stabilization, balloon volume was adjusted to achieve an LV end-diastolic pressure (LVEDP) of 8 to 9 mmHg and held constant during the protocol. LV-developed pressure (DevP) was calculated as: DevP = LV systolic pressure (LVSP) – LVEDP. The rate pressure product (RPP = DevP x heart rate) was calculated to estimate the work performed by LV. The perfused hearts were placed in a 20 mm glass tube in a 9.4 T vertical bore magnet and maintained at 37°C allowing for simultaneous measurement of LV contractile function and HEP.[16-18] The left ventricular wall stress was determined by using the law of Laplace: wall stress = (left ventricular systolic pressure x ventricular radius)/(2 x ventricular wall thickness), as described previously.[15] HEP were measured by ^{31}P NMR spectroscopy using a Varian VNMRS spectrometer (9.4T,161.4 MHz).[16,18] Each ^{31}P-NMR spectrum resulted from the average of 240 free induction decay signals over 10 min. Fully relaxed spectra acquired with a recycle time of 20 s were used to

determine saturation factors. Intracellular pH was calculated from the chemical shift of intracellular inorganic phosphate (Pi) relative to PCr.[16,18,19] At the end of each experiment, beating hearts were freeze-clamped using Wollenberger tongs pre-cooled in liquid nitrogen and stored at −80 °C for subsequent analysis of total creatine concentration by high-performance liquid chromatography (HPLC).[47] The tissue water content was assessed by drying a tissue powder aliquot for 72 h at 75°C. Thereafter, the cytosolic creatine concentration ([Cr]) was calculated as the difference between total creatine concentration [Cr_{total}] measured by HPLC[47] and [PCr] measured by ^{31}P NMR. The free [ADP] was calculated using the creatine kinase reaction (Equation 1) assumed to be at equilibrium, where Keq = 1.66×10^9 M^{-1}.[16,18]

Equation 1: $[ADP] = ([ATP][Cr])/([PCr][H^+])Keq$

The free energy of ATP hydrolysis ($\Delta G_{\sim ATP}$) was calculated by using the Equation 2, where ΔG^0 (−30.5 kJ/mol) is the value of $\Delta G_{\sim ATP}$ under standard conditions, R = 8.314 J/mol·K, and T = 310 K. The value of $\Delta G_{\sim ATP}$ is negative, which denotes that the reaction is exergonic or energy-releasing. For the sake of clarity, we use the absolute value of $\Delta G_{\sim ATP}$, i.e. $|\Delta G_{\sim ATP}|$.[16,18]

Equation 2: $|\Delta G_{\sim ATP}| = |\Delta G^0_{\sim ATP} + RT\ ln[ADP][Pi]/[ATP]|$

Magnetization transfer experiments

As per study protocol (Figure 2B), in separate cohort of hearts perfused with 120 nM dobutamine (n=10) or 60 nM TA1 (n=9), the ATP synthesis rates were measured with the 2-site saturation transfer technique by applying a low-power narrow-band radiofrequency

in order to saturate γ-ATP resonance and measure changes in the PCr and Pi resonance.[16-18] Spectra were acquired without (M_0) and with 4.8 s (M_∞) a selective saturating pulse. Each spectrum averaged a total of 256 scans, interleaved between alternating sets of eight M_0 and M_∞ scans. Scans and measurements were acquired in 44 min. During the M_0 acquisition, a same-power radiofrequency pulse was used and targeted at an equal frequency offset downfield from the observed resonance to eliminate direct attenuation of the observed resonance by the γ-ATP targeted saturation pulse; thus, control for "radiofrequency spillover" by a symmetrical irradiation targeted at the same offset on the opposite side of the Pi resonance. The unidirectional pseudo–first-order rate constant of ATP synthesis was calculated using the following formulae: kf = $(M_0/M_\infty)/T1\ M_\infty$ and flux = kf [Pi], where T1 is the intrinsic longitudinal relaxation time for Pi, [Pi] and M_0 and M_∞ are magnetizations of Pi at 0 and 4.8 s, respectively.

Supplemental Tables

	Log $[Ca^{2+}]$ M											
	-3.82362	-4.1831	-4.86012	-5.35655	-5.61979	-5.79588	-6.11526	-6.34814	-6.53955	-6.70841	-6.8636	-8
**100 µM TA1**												
Mean	0.05305675	0.057317	0.06206925	0.064372	0.0570145	0.045375	0.03834	0.031505	0.030946	0.022154	0.023317	0.01998
SEM	0.001496137	0.002385	0.00376765	0.00094008	0.00072845	0.001724	0.000635	0.001376	0.001572	0.001052	0.00158	0.001061
n	4	4	4	4	4	4	4	4	4	4	4	4
**33.3 µM TA1**												
Mean	0.05026975	0.04867	0.059208	0.06334375	0.05761625	0.046334	0.039298	0.037551	0.029755	0.024675	0.027264	0.021282
SEM	0.003833628	0.001879	0.00131911	0.00161561	0.0004263	0.00295	0.001333	0.001011	0.001521	0.000648	0.0016	0.002073
n	4	4	4	4	4	4	4	4	4	4	4	4
**11.1 µM TA1**												
Mean	0.05523725	0.056824	0.06139275	0.060339	0.0567485	0.050122	0.040685	0.035907	0.034043	0.030898	0.026254	0.025494
SEM	0.001251529	0.001196	0.0028695	0.00143004	0.00211046	0.002071	0.001884	0.001556	0.000944	0.003467	0.000702	0.004049
n	4	4	4	4	4	4	4	4	4	4	4	4
**3.70 µM TA1**												
Mean	0.0499105	0.05308	0.058107	0.0604795	0.05463025	0.0505	0.03516	0.029479	0.027932	0.023028	0.021999	0.022396
SEM	0.001593899	0.001492	0.00140628	0.0002443	0.00078079	0.002338	0.001527	0.001309	0.00117	0.00241	0.000805	0.002591
n	4	4	4	4	4	4	4	4	4	4	4	4
**1.23 µM TA1**												
Mean	0.0493245	0.049426	0.05509775	0.0535935	0.048719	0.041392	0.026026	0.018918	0.022058	0.018714	0.017679	0.01661
SEM	0.002179618	0.001393	0.00195727	0.00087281	0.00112854	0.001874	0.001627	0.001724	0.002011	0.002222	0.000879	0.000991
n	4	4	4	4	4	4	4	4	4	4	4	4
**0.412 µM TA1**												
Mean	0.04359125	0.046339	0.051047	0.0497745	0.04478575	0.03376	0.017133	0.015404	0.018278	0.015726	0.012809	0.013979
SEM	0.002099976	0.001097	0.0017987	0.00224444	0.0018472	0.002159	0.000734	0.001457	0.001165	0.001515	0.003643	0.001359
n	4	4	4	4	4	4	4	4	4	4	4	4
**0.137 µM TA1**												
Mean	0.0436085	0.042302	0.04377525	0.0406995	0.02888725	0.027861	0.014349	0.011837	0.013036	0.013287	0.013169	0.012478
SEM	0.001250243	0.001109	0.00227676	0.00253388	0.00111286	0.000871	0.00065	0.001081	0.001709	0.00247	0.001699	0
n	4	4	4	4	4	4	4	4	4	4	4	4
**0 µM TA1**												
Mean	0.03365225	0.036152	0.039974	0.031646	0.021482	0.015707	0.012013	0.008938	0.009622	0.01154	0.010445	0.008367
SEM	0.00119867	0.001211	0.00085971	0.00204072	0.00213563	0.002155	0.000995	0.001484	0.001653	0.000976	0.002639	0.001513
n	4	4	4	4	4	4	4	4	4	4	4	4

Supplemental Table I. Fitted data for Figure 1B.

	Log [Ca^{2+}] M						
	0.000000001	0.000001	0.00000178	0.00000316	0.00000562	0.00001	0.0001
**Vehicle**							
Mean	0.00015525	0.002819	0.09058125	0.32620975	0.5932115	0.797321	1
SEM	0.000120145	0.005691	0.03189915	0.03280231	0.03048752	0.020625	0
n	4	4	4	4	4	4	4
**1 µM TA1**							
Mean	0.01840375	0.202774	0.428477	0.6396475	0.8971645	1.167791	1.347448
SEM	0.014116053	0.021522	0.02644169	0.03393381	0.06188654	0.052743	0.070347
n	4	4	4	4	4	4	4
**10 µM TA1**							
Mean	0.129558	0.942761	1.378025	1.530404	1.5500623	1.562846	1.556041
SEM	0.003352093	0.091482	0.0888531	0.10596397	0.11127375	0.11246	0.110777
n	3	3	3	3	3	3	3

Supplemental Table II: Fitted data for Figure 1D.

Timepoint (min):	5	15	25	35	45	55	65	75
**Fig 3_TA1**	15	15	14	14	15	15	15	15
**Dob**	11	11	10	10	11	11	11	9
**Fig 4_TA1**	15	15	14	14	14	14	15	14
**Dob**	11	11	10	10	10	10	11	11
**Fig 5A_TA1**	15	15	14	14	14	14	15	14
**Dob**	11	11	10	10	10	10	11	11
**Fig 5B_TA1**	15	15	14	14	14	14	15	14
**Dob**	11	11	10	10	10	10	11	9

Supplemental Table III. Number of animals for all timepoints in Figures 3, 4 and 5.

Supplemental Figures and Figure Legends

Supplemental Figure I. TA1 increases left ventricular systolic pressure (LVSP) and preserves ATP, pH and total creatine concentration [Cr_{total}]. A, LVSP increased progressively with increasing concentrations of both TA1 and dobutamine. **B-D,** [ATP] (**B**) and pH **(C**) underwent minor changes throughout the protocol and were similar in both TA1 and dobutamine groups. Intracellular [Cr_{total}] (**D**) was unaffected by both TA1 and dobutamine. Dob indicates dobutamine. N = 15 (in TA1 group) or 11 (in dobutamine group). Data shown are mean ± SEM. Averages of the two-time-point measurements per substance concentration were compared. P-values were obtained by paired t-tests (baseline vs. incremental concentrations of dobutamine or TA1) and unpaired t-test (60 nM TA1 vs. 120 nM dobutamine). **** indicates P<0.0001 vs. TA1-zero baseline. ††, †††, and †††† indicate P<0.01, P<0.001, and 0.0001 vs. dobutamine-zero baseline, respectively.

Supplemental Figure II. Equivalent dose of TA1 (60 nM) achieves higher left ventricular systolic pressure (LVSP) and similar rate constant for ATP synthesis and ATP flux compared with 120 nM dobutamine. A, During magnetization transfer, 60 nM TA1 achieved higher LVSP compared to 120 nM dobutamine. **B and C**, Rate constant for ATP synthesis (**B**) and ATP flux (**C**) were similar between 60 nM TA1 and 120 nM dobutamine groups. Dob indicates dobutamine. N = 9 (in 60 nM TA1 group) or 10 (in 120 nM dobutamine group). Data shown are mean ± SEM. P-values were obtained by unpaired t-tests. ** indicate P<0.01.

Supplemental Figure I

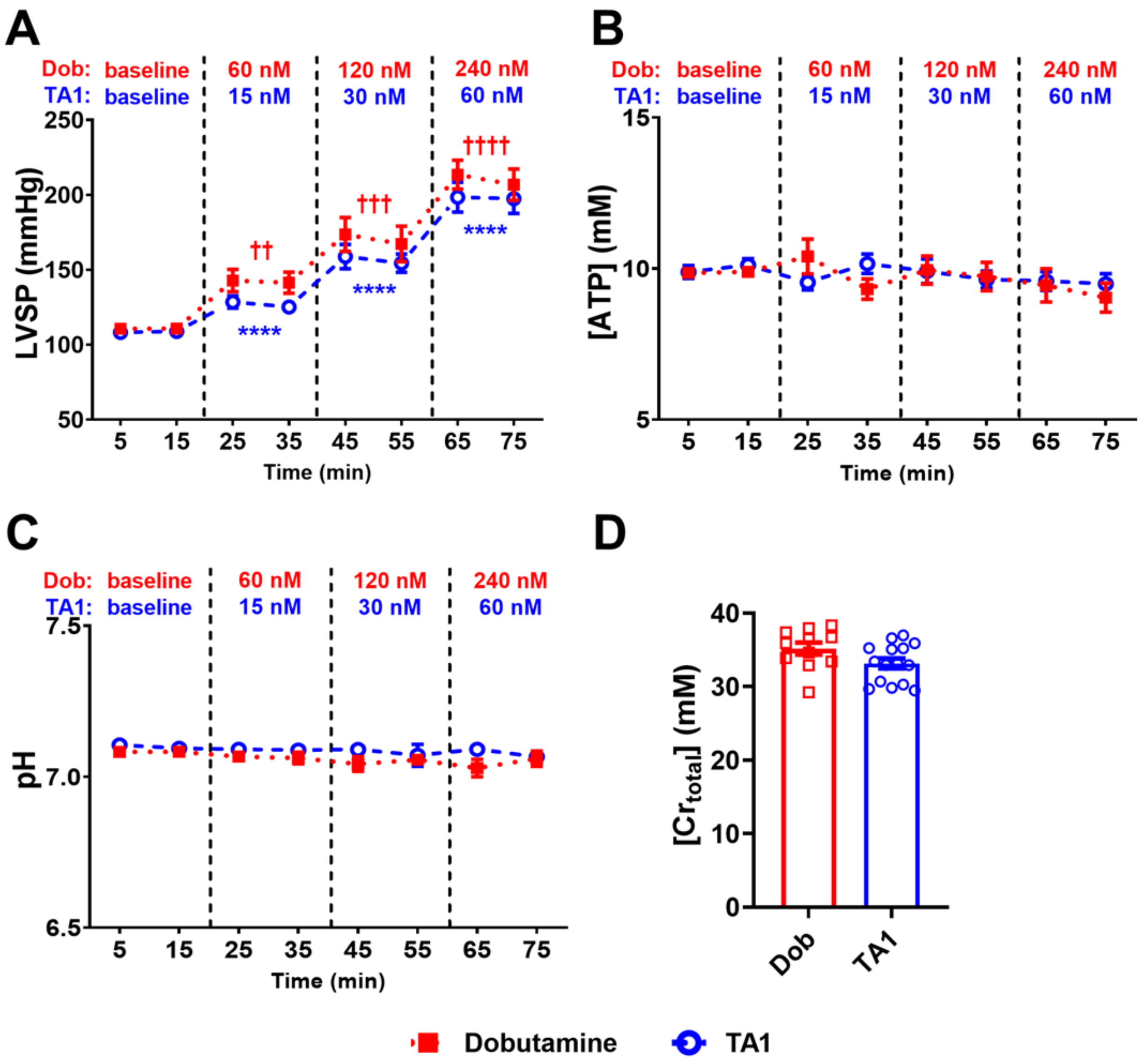

Supplemental Figure II

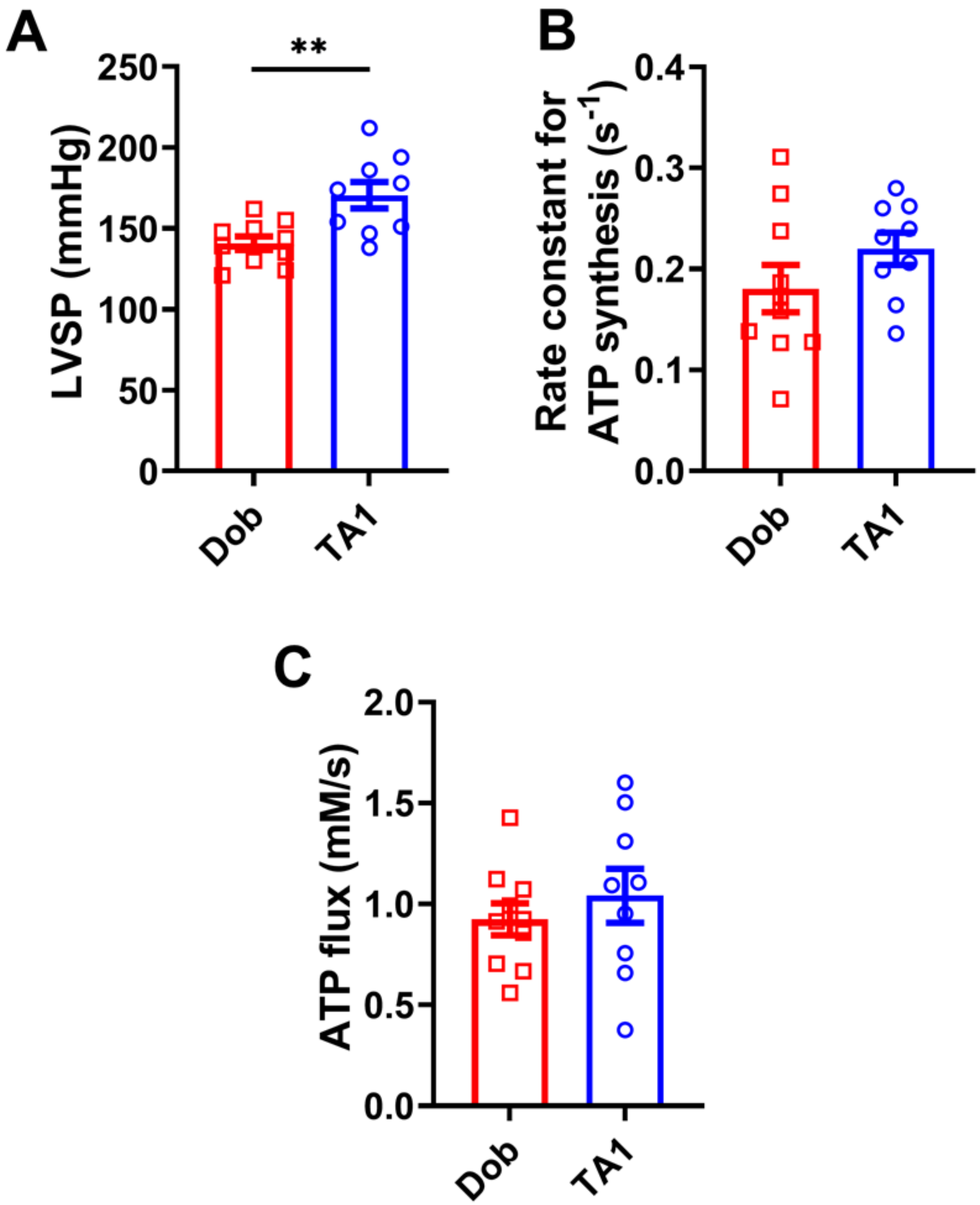

Supplement: Supplementary file 1 [file hhf-15-e009195-s001.pdf]
